# A Fog Computing Enabled Virtual Power Plant Model for Delivery of Frequency Restoration Reserve Services

**DOI:** 10.3390/s19214688

**Published:** 2019-10-28

**Authors:** Claudia Pop, Marcel Antal, Tudor Cioara, Ionut Anghel, Ioan Salomie, Massimo Bertoncini

**Affiliations:** 1 Computer Science Department, Technical University of Cluj-Napoca, Memorandumului 28, 400114 Cluj-Napoca, Romania; claudia.pop@cs.utcluj.ro (C.P.); tudor.cioara@cs.utcluj.ro (T.C.); ionut.anghel@cs.utcluj.ro (I.A.); ioan.salomie@cs.utcluj.ro (I.S.); 2 R&D Department, Engineering and Ingegneria Informatica S.p.A., Via San Martino della Battaglia 56, 00185 Rome, Italy; Massimo.Bertoncini@eng.it

**Keywords:** smart energy grid, virtual power plants, fog computing, modeling, simulation, frequency restoration reserve

## Abstract

Nowadays, centralized energy grid systems are transitioning towards more decentralized systems driven by the need for efficient local integration of new deployed small scale renewable energy sources. The high limits for accessing the energy markets and also for the delivery of ancillary services act as a barrier for small scale prosumers participation forcing the implementation of new cooperative business models at the local level. This paper is proposing a fog computing infrastructure for the local management of energy systems and the creation of coalitions of prosumers able to provide ancillary services to the grid. It features an edge devices layer for energy monitoring of individual prosumers, a fog layer providing Information and Communication Technologies (ICT) techniques for managing local energy systems by implementing cooperative models, and a cloud layer where the service specific technical requirements are defined. On top, a model has been defined allowing the dynamical construction of coalitions of prosumers as Virtual Power Plants at the fog layer for the provisioning of frequency restoration reserve services while considering both the prosumers’ local constraints and the service ones as well as the constituents’ profit maximization. Simulation results show our solution effectiveness in selecting the optimal coalition of prosumers to reliably deliver the service meeting the technical constraints while featuring a low time and computation overhead being feasible to be run closer to the edge.

## 1. Introduction

Traditionally, energy grids are constructed around centralized broadcast-like mono-directional energy systems, where electricity is remotely generated by power plants and transported over complex networks and infrastructure to the consumption points, with significant costs for interconnecting remote areas. This model is cost effective up to a point and fits well with cloud based management infrastructure. However, with the growing deployment of small scale prosumers such as combined heat and power plants, distributed energy generation units, electric cars, and batteries, the architecture of grid systems needs to be decentralized to overcome the increasing complexity and new challenges of energy management operations. The first challenge is the efficient integration of new deployed small scale intermittent renewable energy sources (RES) while preserving continuity and security of energy supply. In this case if the locally generated renewable energy is not self-consumed, then local problems such as overvoltage, losses, and lifetime decay affecting the transformers and electric equipment may appear at the local microgrid level and could be escalated to higher management levels. The second challenge is related to the fact that centralized energy systems do not consider the local conditions, making it difficult to be adapt and optimize them to meet user needs, and meaning they do not provide incentives for prosumers to reduce/increase their demand by shifting flexible energy. The third one is the rather high limits for accessing the energy markets and also for the delivery of ancillary services, which constitute a barrier hindering small scale prosumer participation. More significant energy and flexibility may be offered and delivered to system operators in their respective markets, if it is aggregated at scale driving the implementation of local cooperative models.

In this context, parts of the energy system are transitioning towards more diverse cooperative and decentralized sub-systems, where energy management may effectively take place at the local level by coordinating small scale prosumers to offer valuable services as a whole to the main grid, thereby avoiding the need for additional grid reinforcement and facilitating efficient grid balancing. As a result, local energy systems have been increasingly researched in the last few years. State of the art literature [1,2,3,4] converge towards the decentralized local-level coordination and management of local energy resources, which are located within a well-defined limited geographical boundary and include a variety of local electricity (RES or conventional generation), energy storage and/or flexible loads, including EVs and cross-resource integration. Also, several models have been defined for realizing the decentralized coordination of local energy resources, such as prosumer community groups, energy cooperatives, Virtual Power Plants (VPPs), [5] and multi-energy hubs [6,7]. 

The main technological challenge that limits adoption of such models is the is the lack of finer grained ICT infrastructure for enabling near real time scalable decentralized management and operational control of the local energy systems. Cloud based solutions are not effective in this case, especially for the provisioning of near real time ancillary services (such as frequency restoration reserve) as this kind of infrastructure features high latency associated with centralization, meaning it is unable to consider localized constraints and conditions. Usually in this case all the energy data collected from monitoring devices are sent to the cloud for processing, thus generating high costs in terms of bandwidth, storage and low reactivity. To address these issues, fog computing infrastructure has been proposed, encouraging the processing of monitored data closer to the edge components [8,9]. In addition, in the context of smart energy grid management, very few approaches may be found in literature, with most of them providing general architecture and failing to identify what kind of energy analytics can be shifted towards the edge, especially for near real time ancillary services. 

In this paper, we address the on the fly construction of VPPs, which usually combine and coordinate local energy production sources with energy storage systems and flexible assets featuring controllable loads, to deliver frequency restoration reserve service. Such local coordination helps to utilize the full potential of the decentralized energy systems through the use of local resources and wider engagement of prosumers, regardless of their scale, in energy management processes. We propose a fog based infrastructure and model allowing the aggregation of energy resources in VPPs to deliver frequency restoration reserve services to the main grid while increasing the profit and reducing the energy costs for the constituent members. As result, the local and grid level value streams can be combined to obtain a more profitable and optimal management of local energy systems, while actively contributing to the energy grid sustainability objectives.

The main contributions of this paper are the following:

- The definition of a decentralized infrastructure for smart energy grid management featuring a hierarchy of three layers: the energy monitoring edge devices layer, the fog layer providing ICT resources for managing local energy systems to deliver near real time ancillary services for the main grid, and the cloud layer including the specific services for the management of the whole distribution energy system;
- A VPP model for dynamically construction of prosumer coalitions at the fog layer that might be extended and customized to the specificity of the ancillary service type to be delivered, while maximizing the profit of the prosumers.
- A model extension for frequency restoration reserve service provisioning based on constraints satisfaction of local prosumers and mixed integers non-linear optimization.

The rest of the paper is structured as follows: Section 2 details the state of the art processes regarding VPPs and decentralized energy grid management, Section 3 presents the proposed fog based architecture and VPP model, Section 4 describes the model extension for the provisioning of frequency restoration reserve service, Section 5 presents simulation based results considering a relevant data set, while Section 6 concludes the paper.

## 2. Related Work

The state-of-the-art literature investigation conducted showed that only a few approaches are addressing VPP modeling, creation, and optimal decentralized management in relation to different smart energy grid sustainability objectives [10,11,12,13]. Most of the literature approaches address day-ahead or intra-day markets which feature more relaxed time constraints, with their associated optimization problems being solved under the latency of cloud-based solutions.

In reference [14], VPPs are constructed and managed to minimize the operational cost of their constituent energy resources while considering the energy loss and energy price for the day-ahead. The optimization problem is formalized as a constraint satisfaction problem which is solved using an Imperialist Competitive Algorithm under technical constraints. In reference [12], the VPP day-ahead and intra-day optimal generation schedule is addressed in relation with Demand Response (DR) programs. The stochastic parameters of the optimization problem considered are the forecasted wind energy production and energy price. A profit based VPP scheduling model is proposed using the Conditional Value-at-Risk [15] as a form of risk management in decision making. The solution is effective in providing the feedback needed for DR programs selection, with the results showing a 30% improvement of VPP profit. Similarly, stochastic programming models for optimal VPP participation in the day-ahead energy market and intra-day market are proposed in references [16,17,18]. The authors model the uncertainty in energy prices, generation, and consumption by using different risk management strategies: conditional value at risk, second-order stochastic dominance constraints, and the Point Estimate Method. In reference [19], the problem of trading the VPP’s aggregated energy to maximize the expected profit is modeled as a two-stage stochastic mixed-integer linear programming model. The results show that the proposed model can maximize the VPP’s short term profit when most of the energy trading decisions take place in the day-ahead market. In a similar manner, the energy aggregators’ opportunities to manipulate the energy price in electricity markets are discussed in detail in references [20,21]. The authors study the problem of estimating the profit that an aggregator may obtain and show that even if it is computationally difficult, efficient algorithms exist when the topology of the network is acyclic. In reference [22], the authors analyze the aggregation of stochastic and deterministic renewable energy sources in a VPP to reliable generate energy which can be traded in the European Power Exchange (EPEX)/European Energy Exchange (EEX) using existing market products, while in reference [23], novel decentralization scenarios like vehicle to grid are investigated and an energy trading framework is proposed based on blockchain, contract theory, and edge computing.

The decentralized VPP optimization and coordination is only partially addressed and discussed. A VPP construction model leveraging on decision area variables is proposed in reference [24], aiming to establish a distributed coordinated control of the distributed energy resources. Regional load density, power consumption levels, administrative ranks, economic levels, and user importance are considered as criteria for determining the VPP decision area. An architecture which aims to aggregate distributed energy resources with the physical domain limited to single Points of Delivery of the distribution network is introduced in reference [25]. The advantage brought about by this approach is the level of decentralization, with the control being moved to the energy resources side. In reference [26], a critical review of literature approaches in relation to VPP and multi-energy systems is conducted. The authors propose the adoption of holonic energy systems as a new management paradigm targeting efficient decentralization through adaptive control topologies and demand responsive energy management while adding features such as local autonomy and global energy balance. The authors of reference [27] identify the need for decentralized decision support systems for the VPP coordinators which have to consider huge amounts of data such as individual resource thermal [28] and electrical power production, size, efficiency, typology, and remuneration. VPP coalitions of wind generators and electric vehicles are considered in reference [29]. The vehicles are modeled using distributed software agents and used as energy storage devices. 

Some approaches are leveraging on game theory to address the problem of optimal energy distribution by dynamically changing the size of the coalition of prosumers [30,31]. An optimal coalition formation mechanism of distributed energy sources using a game theoretical perspective is described in reference [32]. A hierarchical coalition formation is proposed to achieve a state of cooperative equilibrium among the distributed energy sources while providing the best possible response to Distributed System Operator (DSO) requests. The authors show that their proposed scheme provides optimal outcomes and it is scalable enough to participate in real-time operation. In reference [16], the authors propose the use of cooperative game theory approaches to split and allocate a VPP’s profit among the aggregated distributed energy resources. Similarly in reference [33], a methodology for creating coalitions of distributed generation units based on game theory is proposed. It features a classification model of distributed energy resources considering fourteen parameters including technical, economic and behavioral ones. The VPPs constructed in this way can participate in demand response programs in both Medium and Low Voltage distribution networks. Finally, in reference [31], a game-theoretic peer-to-peer energy trading scheme is developed, with the authors showing that the coalition among different prosumers is a stable coalition, and managing to reduce the cost of energy by more than 25%.

In reference [34], the authors investigate the possibility of applying edge computing principles for smart grid management and identify challenges and open issues in this context. By using a distributed decentralized architecture provided by the edge computing paradigm, scheduling and fault tolerance aspects of a smart grid can be addressed while also solving problems related to distributed generators and prosumers interaction. Similarly, in reference [35] an analysis of edge computing solutions for the smart grid is conducted. The authors focus on modeling smart homes integration in smart grids when dealing with a limited power budget, highlighting privacy as the main advantage. An approach for time-series analytics with the edge computing applied to the smart grid and manufacturing industries is presented in reference [36], but the focus lies more on integrating the wired/wireless communication networks that will use real time computing at the edge of the network. Reference [37] describes the energy lattices as fog computing architecture applied for smart grids. In this architecture, devices such as smart meters and micro-grids will achieve energy efficient management using availability and price as the main criteria. Authors of reference [8] discuss the existing fog computing approaches and identify important functionalities for such platforms in the area of sustainable smart cities. The smart power grid is identified as an important use case for fog computing especially for reducing operational costs, integrating renewable energy systems, balancing the system, and monitoring power generation, demand, and storage. In reference [38], an energy management-as-a-service over fog computing platform is introduced for both home energy management and microgrid-level energy management. The approach uses low-power and low-cost devices for computation, storage, and communication to build a service oriented fog computing platform. In reference [39], an arbitrage strategy is defined for VPPs participation in ancillary services market targeting the spinning reserve and frequency restoration reserve services. The optimization model considers the supply–demand balancing, transmission network topology, and security targeting the VPP’s profit maximization. In reference [40] a two-stage stochastic programming approach is used to address the problem of VPP trading in a market of ancillary services. It incorporates a risk-averse optimal offering model based on conditional value-at-risk while considering the uncertainty regarding energy generation/consumption and energy prices in balancing markets.

By analyzing the state of the art research, it can be seen that there are few approaches addressing the problem of dynamic construction of coalitions of energy resources to provide management services for energy players such as the DSO. They are not considering the fog infrastructure for addressing the VPP management and optimal construction problem as close as possible towards the edge components. Also, the localization and near real time constraints of specific type of ancillary services such as frequency restoration reserve is not properly addressed. The solution proposed in this paper goes beyond the state of the art research by providing a generic VPP construction and optimization model over a fog computing based infrastructure, allowing the definition, formalization, and provisioning of near real time frequency restoration reserve service as close as possible to the edge. Regarding VPPs and decentralized management of frequency restoration reserve, we could not identify any relevant state-of-the-art approach addressing it by using a fog computing based infrastructure. 

## 3. Fog Based VPP Model

**Figure 1:**
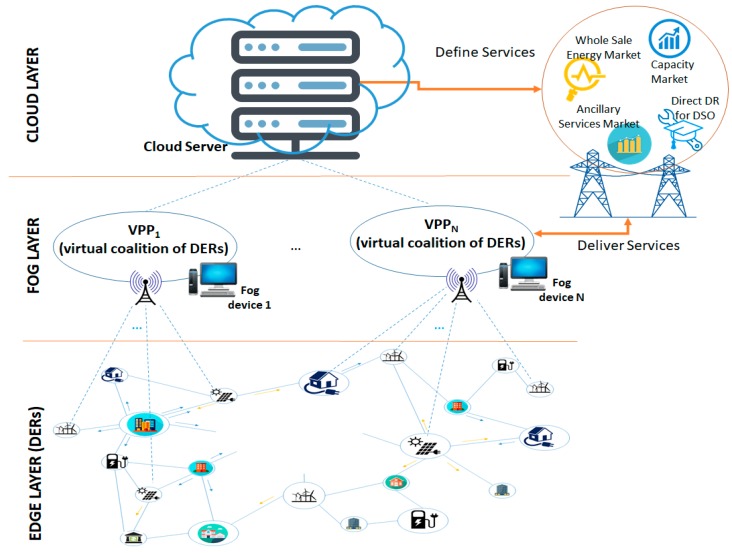
Fog computing and Virtual Power Plant (VPP) model in smart grids.

The proposed model is based on a hierarchy of three layers (Figure 1):

- An edge layer contains the physical IoT energy metering devices associated with each individual prosumer (or Distributed Energy Resource (DER)) from the smart grid;
- A fog layer contains computational resources associated with a local geographical area (i.e., the microgrid area) enabling the construction of virtual coalitions of prosumers in VPPs to provide various services for the main grid, while maximizing the revenue of the local constituent energy resources;
- A cloud layer represented by cloud server on which the DSO runs its analytics to define specific services for optimal management of grid resources, which can be addressed by an individual prosumer (if it is large enough) or by a virtual coalition of prosumers.

This approach provides the following advantages:

- Proximity to the prosumers—the analytics for solving the optimization problems are run closer to the prosumers, thus minimizing the decision time and providing the opportunity for selecting prosumers to participate in VPP coalitions regardless of their size or scale;
- Increased Locality—the virtual coalitions can be associated with a local microgrid and, as a result, locally address the potential management problems and avoid their escalation to higher grid levels;
- Reduced Latency—in the data traffic from the lower edge level to the higher fog level (where the VPPs are constructed) and vice-versa. This is also an important condition of the availability of a high amount of data from the smart meters.

**Figure 2:**
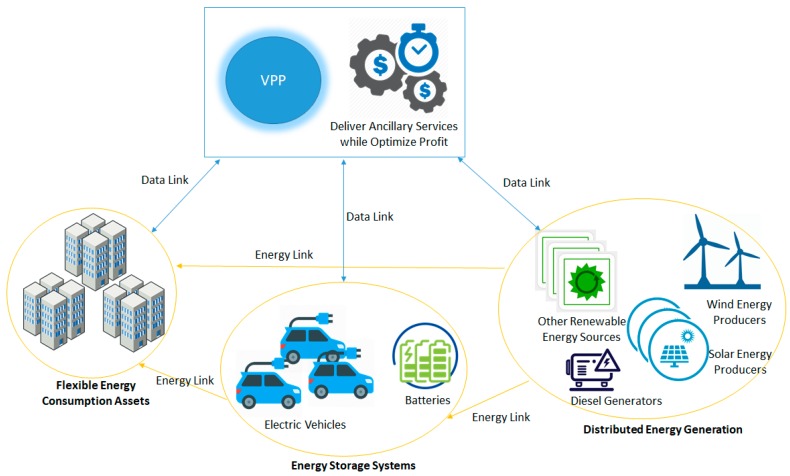
The VPP model considered.

In the next paragraphs, we provide the underlying model to create the fog level dynamic coalitions, which will aggregate in VPPs the following types of prosumers (see Figure 2):

- Distributed Energy Generators (DEG) such as small-scale wind power plants, photovoltaic units, Combined Heat and Power (CHP) systems, or diesel generators;
- Energy Storage Systems (EES), such as batteries or UPSs;
- Flexible Energy Demand Assets (FDA).

The prosumer is modeled as a tuple consisting of its predicted energy profile over a future time interval T in which the VPP is constructed and the prosumer type is:

(1) $$Prosumer\left[k\right]=\left(E_{k}\left[T\right],type=\left\{DEG,\;EES,FDA\right\}\right).$$

The energy profile of the prosumer is represented as a set of energy values sampled at equidistant time stamps during the time interval T over which the VPP coalition is created to provide a specific service:

(2) $$E_{k}\left(T\right)=\left\{E_{K}\left(i\right)|i\in \left\{0\ldots T\right\},\;k\in \left\{1\ldots N\right\}\right\}.$$

We consider that the local grid includes N prosumers of different types and energy profiles, each of them having their own specific local constraints which need to be met:

(3) N=C+P+S

where C is the number of flexible energy assets, P is the number of energy producers, and S is the number of energy storage devices. 

The goal of the coalition of prosumers construction process is to select a subset of the energy prosumers from the local grid portfolio which best fulfils the optimization objectives defined for the type of ancillary service that needs to be delivered by the VPP, while meeting each energy prosumer local constraints and maximizing their profit. The VPP coalition is represented as a binary array of length N, where a value of 0 on position k means that prosumer k is not part of the coalition, while a value of 1 means that the prosumer is included (taken) into the coalition.

(4) $$\begin{array}{c} VPP=\left\{\left(taken_{k},Prosumer\left[k\right]\right)|k\in \left\{1\ldots N\right\},\;taken_{k}\in \left\{0,1\right\}\right\}\\ taken_{k}=\{\begin{array}{c} 0,Prosumer\left[k\right]\;is\;not\;part\;of\;the\;VPP\\ 1,Prosumer\left[k\right]\;is\;part\;of\;the\;VPP \end{array} \end{array}$$

The search space of the optimization problem is $2^{N}$ where the set of all subsets that can be formed has the elements of a set of cardinality N, thus making the search problem NP-complete. 

In the following, each prosumer type, as VPP main constituents, will be represented at the Fog Level in terms of their parameters and operational constraints.

The *Distributed Energy Generators* are modeled by the following parameters and local operational constraints:

- $E_{K}$—the forecasted energy generation values;
- $U_{L},U_{H}\in \left[0,1\right]$—the lower and upper levels of uncertainty considered in the forecasting process;
- $E_{MAX}^{generation}$ —the maximum energy generation;

The lower and upper limit of uncertainty give the lower and upper bounds of the energy predictions considering the potential prediction errors reported to the actual value that will be monitored in the future:

(5) $$U_{L}*E_{K}\left(i\right)\leq E_{K}^{uncerntainty}\left(i\right)\leq U_{H}*E_{K}\left(i\right)\leq \;E_{MAX}^{generation},\;\forall i\;\in \left\{0\ldots T\right\}$$

The total energy generated by the producers selected in a VPP can be computed as the sum of each individual prosumer energy generation:

(6) $$E_{VPP}^{generation}\left(t\right)=\overset{P}{\underset{k=1}{\sum }}taken\left(k\right)*E_{K}^{uncerntainty}\left(t\right)$$

Furthermore, the coalition is created by considering the risk management in the optimization decision making generated by the uncertainty in the energy generation forecasting processes. This is computed as the weighted difference (ρ) between the forecasted value of the prosumer energy profile $E_{K}$ and the actual values during delivery and represents the cost function in the optimization problem. When the difference is high, the probability of not meeting the forecasted energy values increases, thus increasing the risk of not being able to supply the energy desired, directly impacting the value of profit estimated:

(7) $$risk_{uncertainity}=\rho *\overset{N}{\underset{k=1}{\sum }}\overset{T}{\underset{t=1}{\sum }}\left|E_{K}^{uncerntainty}\left(t\right)-E_{K}\left(t\right)\right|$$

The *Energy Storage Sources*, is modeled by the following parameters and local operational constraints:

- Maximum capacity: $MAX_{K}^{Load}\left[kWh\right],\;k\in \left\{1\ldots S\right\}$
- Depth of Discharge: $DoD_{k},\;k\in \left\{1\ldots S\right\}$
- Initial state: $ESS_{k}^{init}\left[kWh\right]\;,\;k\in \left\{1\ldots S\right\}$
- Maximum Charging Rate on time interval: $MAX_{K}^{Charge}\left[kWh\right],\;k\in \left\{1\ldots S\right\}$
- Maximum Discharging Rate on time interval: $MAX_{K}^{Discharge}\left[kWh\right],\;k\in \left\{1\ldots S\right\}$
- Actual Charging Rate on a time interval: $C_{ESS}^{k}\left[kWh\right],\;k\in \left\{1\ldots S\right\}$
- Actual Discharging Rate on a time interval: $D_{ESS}^{k}\left[kWh\right],\;k\in \left\{1\ldots S\right\}$
- Actual Loaded Capacity: $ESS_{k}\left[kWh\right],\;k\in \left\{1\ldots S\right\}$
- Charge loss factor: $\varphi_{C}\in \left[0,1\right]$
- Discharge loss factor: $\varphi_{D}\in \left[0,1\right]$
- Discharge Cost per unit: $COST_{k}^{D}\left[\frac{€}{\left[kWh\right]}\right]\;,\;k\in \left\{1\ldots S\right\}$
- Charge Cost per unit: $COST_{k}^{C}\left[\frac{€}{\left[kWh\right]}\right]\;,\;k\in \left\{1\ldots S\right\}$

The battery actual loaded capacity must be bounded by the maximum capacity and by the depth of discharge ($DoD_{K}$). Furthermore, the charge and discharge values also must be bounded.

(8) $$DoD_{K}*MAX_{K}^{Load}\leq ESS_{k}\left(t\right)\leq MAX_{K}^{Load}\;k\in \left\{1\ldots S\right\},t\in \left\{1\ldots T\right\}$$

(9) $$0\leq C_{ESS}\left(t\right)\leq MAX_{K}^{Charge},\;k\in \left\{1\ldots S\right\},t\in \left\{1\ldots T\right\}$$

(10) $$0\leq D_{ESS}\left(t\right)\leq MAX_{K}^{Discharge},\;k\in \left\{1\ldots S\right\},t\in \left\{1\ldots T\right\}$$

(11) $$ESS_{k}\left(0\right)=ESS_{K}^{init},\;\forall k\in \left\{1\ldots S\right\},t\in \left\{1\ldots T\right\}$$

When the battery is discharged over a time interval with $D_{ESS}^{k}$ kWh, its actual loaded capacity decreases with $\left(\varphi_{D}+1\right)*D_{ESS}^{k}$, due to the discharge losses. Furthermore, when a battery is charged, the actual loaded capacity increases with $\left(1-\varphi_{C}\right)*C_{ESS}^{k}$, due to the charging losses. A battery cannot be charged and discharged simultaneously.

(12) $$ESS_{k}\left(t\right)=ESS_{k}\left(t-1\right)+\left(1-\varphi_{C}\right)*C_{ESS}^{k}\left(t\right)-\left(\varphi_{D}+1\right)*D_{ESS}^{k}\left(t\right),k\in \left\{1\ldots S\right\}$$

(13) $$C_{ESS}^{k}\left(t\right)*D_{ESS}^{k}\left(t\right)=0,\;k\in \left\{1\ldots S\right\},t\in \left\{1\ldots T\right\}$$

The charge and discharge of a battery increase its operating costs due to wear and thus decrease the overall VPP profit. As a result, the operating cost of the battery over a time interval [0…T] is computed as the negative cost due to battery charge and discharge:

(14) $$\begin{array}{c} OP_{cost}\left(C_{ESS}^{k},D_{ESS}^{k},price\right)=\\ \overset{T}{\underset{t=1}{\sum }}(\left(1-\varphi_{C}\right)*C_{ESS}^{k}\left(t\right)*COST_{k}^{C}+\left(\varphi_{D}+1\right)*D_{ESS}^{k}\left(t\right)*COST_{k}^{D})) \end{array}$$

The overall energy charged and discharged by the batteries over a time interval can be computed as the sum of the energy charged or discharged by each individual battery from the grid:

(15) $$C_{ESS}\left(t\right)=\overset{S}{\underset{k=1}{\sum }}C_{ESS}^{k}\left(t\right);\;D_{ESS}\left(t\right)=\overset{S}{\underset{k=1}{\sum }}D_{ESS}^{k}\left(t\right)$$

The overall cost of charging and discharging the batteries over the optimization interval [0…T] is computed as the sum of the costs for each battery usage.

(16) $$OP_{cost}\left(C_{ESS},D_{ESS},price\right)=\overset{S}{\underset{k=1}{\sum }}OP_{cost}\left(C_{ESS}^{k},D_{ESS}^{k},price\right)$$

The reward of operating the batteries selected in a VPP by selling and buying energy from the energy marketplace considering that the energy price is defined as:

(17) $$\begin{array}{c} R_{ESS}\left(C_{ESS},D_{ESS},price\right)=\\ \left(\overset{T}{\underset{t=1}{\sum }}\left(D_{ESS}\left(t\right)-C_{ESS}\left(t\right)\right)*price\left(t\right)\right)-OP_{cost}\left(C_{ESS},D_{ESS},price\right) \end{array}$$

The *Flexible Energy Demand Assets*, are modeled by the following parameters and local operational constraints:

- $E_{K}^{baseline}$—the baseline energy consumption of the flexible asset;
- $APC_{Below}^{flexibility}$—the lower bound of average energy consumption defined as the values of the actual energy measured that are below the baseline;
- $APC_{Above}^{flexibility}$—the upper bound of the average energy consumption defined as the actual energy measured that are below the baseline;
- $E_{MAX}^{flexibility}$—the maximum energy consumption of the flexible asset.

The constraints defined for the flexible assets state the amount of flexibility they might provide either for increasing or decreasing their energy profile, being bounded by their adaptability power curve parameters (above or below):

(18) $$0\leq APC_{below}^{flexible}\leq E_{K}^{uncerntainty}\left(t\right)\leq APC_{above}^{flexible}\leq E_{MAX}^{flexibility}$$

The total energy flexibility that can be potentially supplied by the selected prosumers in a VPP is defined as the sum of the energy profiles of the selected prosumers.

(19) $$E_{VPP}^{flexibility}\left(t\right)=\overset{C}{\underset{k=1}{\sum }}taken\left(k\right)*E_{K}^{uncerntainty}$$

Each different generation type exposes the coalition to various risks due to weather conditions, thus diversity of generation types is an important feature of the coalition. Hence, we define a risk measure to increase the diversity of the prosumers, in terms of type generation, selected in a coalition. We consider the total number of different prosumer types as $VPP_{Types}$, while the number of selected prosumers in a solution is denoted as $VPP_{size}=\sum_{k=1}^{N}taken_{k}$. If each prosumer would be evenly distributed, then the ratio of the total number of prosumers in the VPP ($VPP_{size}$) and the total type of prosumers $\frac{VPP_{size}}{VPP_{Types}}$ should be equal to 1. So, we define the diversity measure as the Euclidean distance between the number for each selected prosumers’ type and the ratio $\frac{VPP_{size}}{VPP_{Types}}$.

(20) $$\begin{array}{c} risk_{diversity}=\\ \sigma *\sqrt{\overset{VPP_{Types}}{\underset{p=1}{\sum }}{\left(\frac{VPP_{size}}{VPP_{Types}}-\overset{N}{\underset{k=1}{\sum }}taken\left(k\right)*\left(Prosumer_{K}.type=p\right)\right)}^{2}} \end{array}$$

## 4. VPP Creation for Frequency Restoration Reserve

We aim at identifying the unused generation capacity that can be activated and aggregated in a VPP to offer frequency balancing by injecting inductive reactive power in the grid and correcting the power imbalances. Thus, the above Fog enabled VPP model is extended to allow the dynamic creation of prosumer coalitions around a point in the local grid where an imbalance of reactive power is identified, such that the new constructed VPP may address locally, in an optimal manner, the reactive power fluctuation, and stabilize the grid voltage.

To create these kinds of coalitions, we have extended the prosumer model to incorporate both active and reactive power components which are correlated through the prosumer power factor PF. The power factor is defined as the ratio between the active and apparent power, and it is a value between 0 and 1 (closer to 1 means less reactive power):

(21) $$Prosumer\left[k\right]=\left(E_{K}^{active}\left[T\right],E_{K}^{reactive}\left[T\right],PF_{TYPE},\left\{DEG,\;EES,FDA\right\}\right)$$

The loads in the Smart Grid can have either a lagging power factor, or a leading power factor:

(22) $$PF^{TYPE}=\left\{lagging,leading\right\}$$

A load that “supplies” reactive power is a capacitive load with a leading power factor, while a load that “consumes” reactive power is an inductive load with a lagging power factor. A leading power factor implies that the reactive component of the power is negative because reactive power is supplied to the circuit and the phase angle in this case is in the fourth quadrant. A lagging power factor means that the reactive component of the power is positive because reactive power is consumed from the circuit, and the phase angle in this case is in the first quadrant. Furthermore, if the resource has a constant power factor, then the bounds are given as equal. Using the general active and reactive power formulas as well as the power factor type (leading or lagging) of a resource, the active –reactive energy relationship determined as the following:

(23) $$E_{K}^{reactive}\left(t\right)=\{\begin{array}{c} -E_{K}^{active}\left(t\right)*\sqrt{\left(\frac{1}{{PF_{K}^{operating}}^{2}}-1\right)},\;if\;leading\;PF\\ E_{K}^{active}\left(t\right)*\sqrt{\left(\frac{1}{{PF_{K}^{operating}}^{2}}-1\right)},\;if\;lagging\;PF \end{array}.$$

The actual operating power factor $PF_{K}^{operating}$ of the prosumer k is limited by the power factor limits:

(24) $$PF_{K}^{leading-MIN}\leq PF_{K}^{operating}\leq PF_{K}^{leading-MAX}$$

(25) $$PF_{K}^{lagging-MIN}\leq PF_{K}^{operating}\leq PF_{K}^{lagging-MAX}$$

The reactive energy in the local grid sums up, and the consumed reactive energy of inductive elements (lagging) cancels the supplied reactive energy of the (leading) capacitive elements:

(26) $$E_{grid}^{reactive}\left(t\right)=\sum_{k=1}^{N}E_{K}^{reactive}\left(t\right).$$

The active energy of the grid can be computed as the sum of the active energy produced, and its absolute values should be equal to the active energy consumed by the grid, to stabilize the frequency.

(27) $$E_{grid}^{active}\left(t\right)=\sum_{k=1}^{N}\left|E_{K}^{active-generation}\left(t\right)\right|=\sum_{k=1}^{N}|E_{K}^{active-demand}\left(t\right)|$$

The power factor over the grid can be computed as the ratio between the reactive energy from the grid and the apparent energy in the grid, and it should be kept constant, at around 0.95.

(28) $$PF_{grid}=\frac{E_{grid}^{reactive}}{E_{grid}^{apparent}}=\frac{E_{grid}^{reactive}}{\sqrt{{\left(E_{grid}^{active}\right)}^{2}+{\left(E_{grid}^{reactive}\right)}^{2}\;\;}}$$

**Figure 3:**
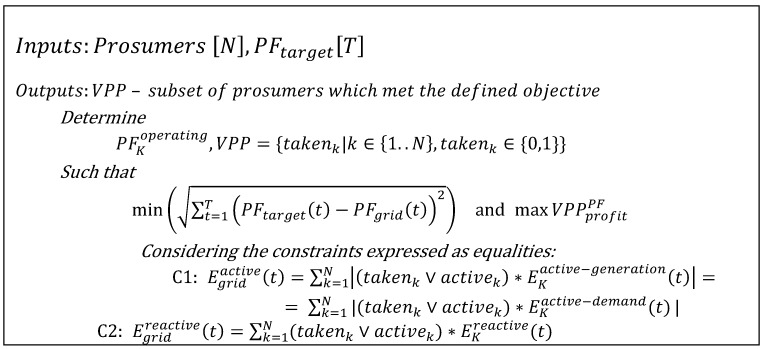

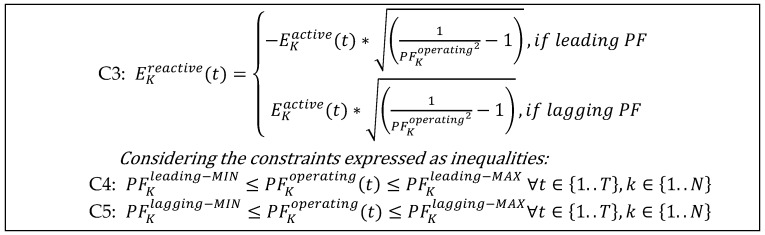
Optimal VPP construction for offering frequency restoration reserve service.

The optimization problem is summarized in Figure 3, and has as inputs the set of prosumers available to be considered in the VPP coalition and the target PF that must be achieved at the local grid level.

The solution of the optimization problem that should be solved at the Fog Level is a subset of energy producers located close to the imbalance point that can compensate for the reactive energy. The optimization objective aims at minimizing the distance between the actual power factor and the target power factor, as well as minimizing the distance between the grid elements that compensate for the imbalance and the imbalance point:

(29) $$\min\left(\sqrt{\overset{T}{\underset{t=1}{\sum }}{\left(PF_{target}\left(t\right)-PF_{grid}\left(t\right)\right)}^{2}}\right)$$

at the same time, the VPP should gain profit by delivering this specific service:

(30) $$\max VPP_{profit}^{PF}=Service_{Reward}-Gen_{cost}$$

## 5. Evaluation Results 

We aim to show the capability of our approach on solving the VPP specific constraints satisfaction problems at the fog level, and by generating the prosumers coalitions at different levels in the hierarchy tailored to technical constraints of different services. 

**Figure 4:**
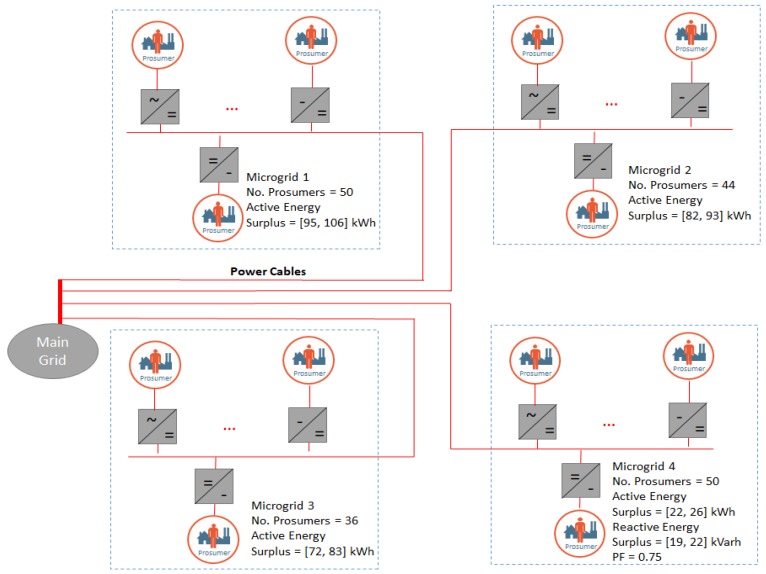
The scenario considered for simulation.

Figure 4 presents the simulated scenario in which we have considered four micro-grids (ids 1 to 4) with a various number of prosumers, the first three with a potential surplus of active energy by activation of additional generation prosumers, while the forth one has a low PF value due to an actual surplus of reactive energy. Table 1 presents the ranges of parameters in which the prosumers part of each microgrid were randomly generated. 

**Figure 5:**
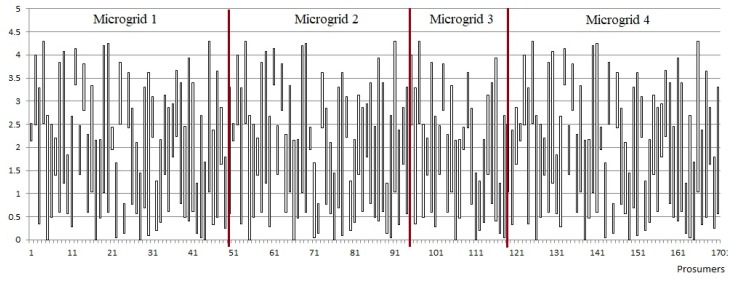
Portfolio of small scale prosumers considered with minimum and maximum energy consumption of each individual prosumer.

Figure 5 presents the pool of prosumers generated and distributed in the 4 micro-grids considered and shown in Figure 4.

We have used the model proposed in Section 3 and Section 4 to address the non-optimal situation generated at the microgrid 4 level. We have considered the provisioning of an automatic Frequency Restoration Reserve (aFRR) service with the technical requirements summarized in Table 2. 

**Figure 6:**
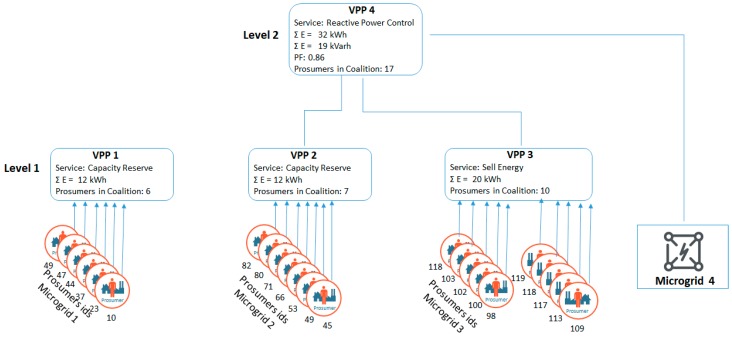
VPP hierarchy constructed using the proposed model.

The optimization problem is trying to identify the prosumers that may be activated to provide additional generation from microgrid 1, 2, and 3 and to aggregate them into VPPs by combining their power profiles such that the surplus of reactive power in microgrid 4 is compensated and the PF improved. At the same time in the construction of VPPs based on additional generation from microgrids 1, 2, and 3, we have considered price signals in the energy market and the provisioning of capacity reserve services. As a result, coalitions of prosumers are constructed in VPPs organized in a two-layer hierarchy (see Figure 6). 

The model optimization problem is a class Mixed Integer Non-Linear Problem (MINLP), because of the integer unknowns represented by the array $taken_{k}$ of length *N*, and the continuous variables represented by the array $PF_{K}^{operating}$ of length T*N. This type of problem is known to be NP-hard [42], thus heuristics and relaxations of the initial problem are used to determine an approximate solution. To solve it we have used a genetic algorithm designed by us specifically for this type of problem, its detailed description is available in reference [43]. In this case the chromosomes are formed by arrays of N multi-genes containing the $taken_{k}$ unknown and the $PF_{K}^{operating}$ power factor profile of the corresponding prosumer *k* within the solution of length *N*. The fitness function is a tuple containing the two-fold objective defined, which is minimizing the distance between the power factor of the VPP and the optimal value and the maximization of constituent’ prosumers profit.

The 1^st^ function using the proposed model VPPs will be constructed for microgrids 1, 2, and 3 with a view of addressing the PF unbalancing by activating a surplus of active energy and selling it on the energy market when the prices are high or by providing reservation capacity on demand. In this way, the surplus of energy is virtually aggregated with a view of increasing the profit of the participant part of the generated coalitions of prosumers. 

**Figure 7:**
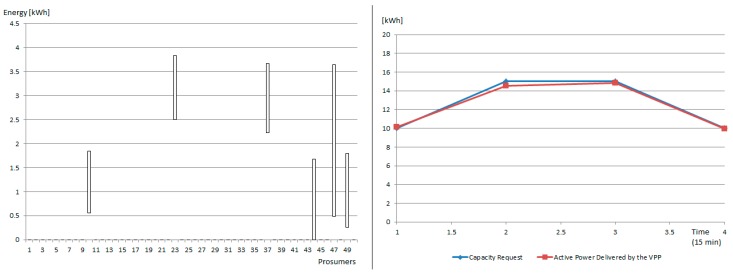
Prosumers in the VPP created in microgrid1: [LEFT] maximum and minimum energy consumption in the service interval and [RIGHT] Requested capacity vs. energy delivered by the VPP.

In the case of microgrid 1, a coalition of 6 prosumers is constructed from the initial pool of 50 prosumers, managing to follow the requested replacement capacity profile with a 97% increase over the one-hour response interval, as shown in Figure 7.

**Figure 8:**
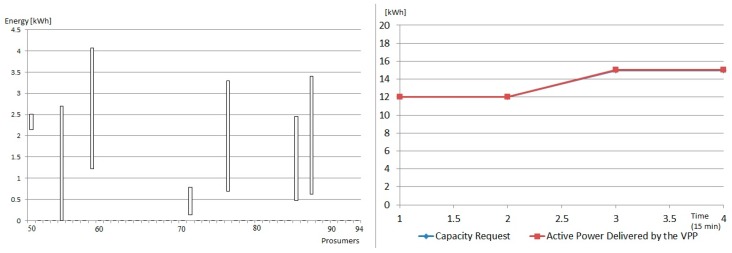
Prosumers in the VPP created in microgrid 2: [LEFT] maximum and minimum energy consumption in the service interval and [RIGHT] Requested capacity vs energy delivered by VPP.

In the case of the microgrid 2, a coalition of prosumers is constructed in a VPP are also able to provide reserve capacity on demand. In this case, eight prosumers from the microgrid are selected and aggregated, being able to provide the requested replacement capacity with an accuracy of 98% (see Figure 8).

**Figure 9:**
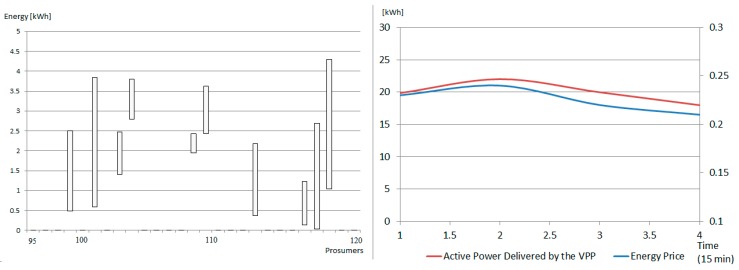
[LEFT] Prosumers selected in the VPP coalition from microgrid3 and [RIGHT] VPP energy generation and energy price in the market.

In the case of the microgrid *3*, the VPP is also created to optimally combine the energy profiles of the energy prosumers grouped in the VPP so that the aggregated amount is proportional to the energy prices from the energy market as depicted in Figure 9 (right). In the first half hour the energy price is high, while during the last half hour, the energy price decreases. Thus, the coalition the prosumers generated will produce more energy in the first part of the optimization interval, leading to an increase of their profit compared to the initial revenue received if they would sell their energy as soon as it is produced. The details of the prosumers selected in the VPP coalition can be seen in Figure 9 (left), while the aggregated energy generation profile of the VPP versus the energy price is shown in Figure 9 (right). The total energy generated by the power plant follows the energy price profile to maximize the profit. A total of 10 prosumers were selected, with an average revenue of 11.25 Euro over a period of one hour, using the energy prices from Figure 9 (right). 

Afterwards, the model will address the low power factor in microgrid 4 by constructing a second level VPP with the aim of virtually aggregating the surplus of active power available at each microgrid level to improve the power factor from the low level of 0.75 and get it as close as possible to the optimal value of 1.

**Figure 10:**
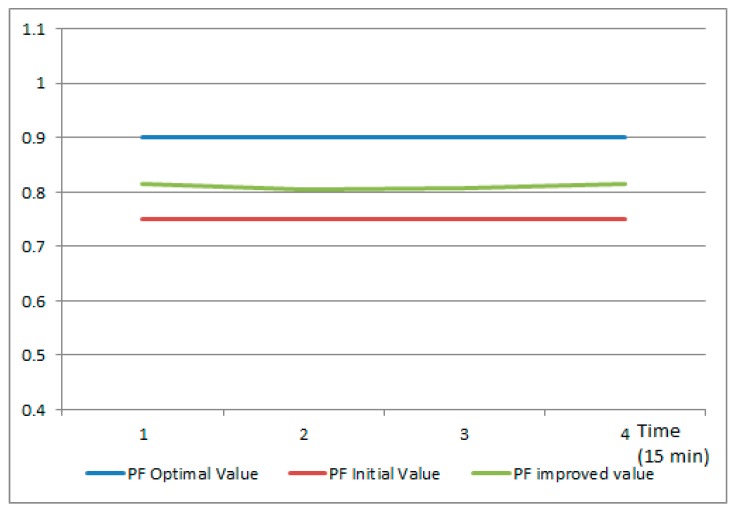
Power factor improvement as result of VPP construction and service delivery.

Figure 10 shows the power factor value in the initial situation (the red line), the optimal value targeted in the frequency restoration reserve service (the blue line) and the PF value after the new second level VPP is created, activated, and used to compensate the reactive power from microgrid 4 (the green line). As a result, the PF value gets close to the optimal value stabilizing the voltage, thus the potential unbalances are locally addressed and is not escalated to the main grid level.

**Figure 11:**
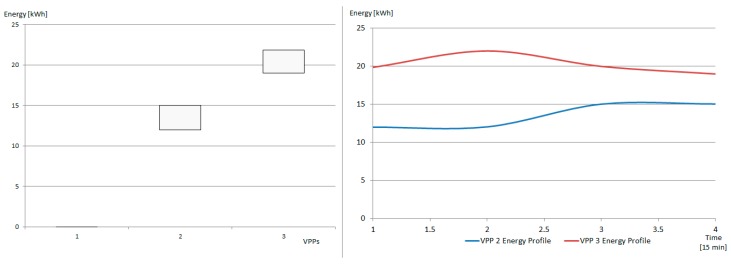
VPPs in the new created hierarchical VPP: [LEFT] maximum and minimum energy consumption in the service interval and [RIGHT] detailed energy profiles.

**Figure 12:**
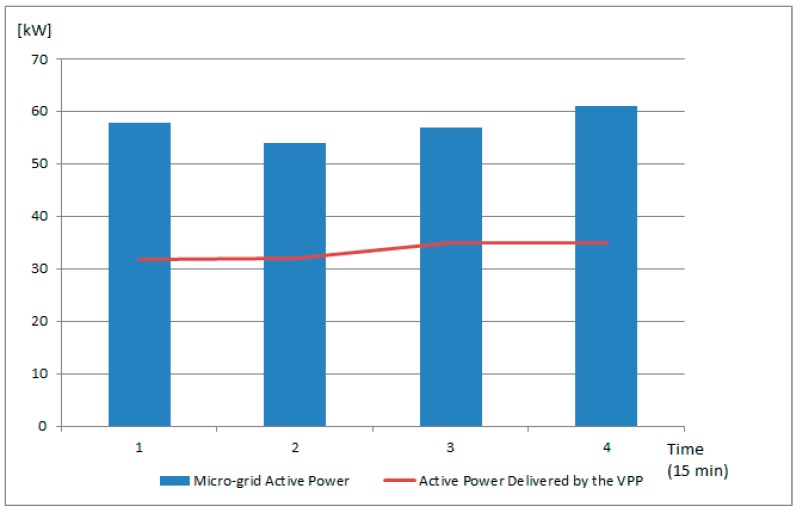
Micro-grid total active power and VPP contributions.

The active power profiles of level one VPP2 and VPP3 (see Figure 6) consisting of 16 prosumers activated from the micro-grids that create the higher level VPP4 to compensate for the low PF of microgrid 4 are shown in Figure 11. Each prosumer has a different PF value, being able to compensate together for the imbalance between the active and reactive power from the grid. The final grid situation is shown in Figure 12. It can be seen that the reactive power has been decreased by adding additional energy prosumers with a leading power factor. Furthermore, the active power in the grid has increased by around 30 kW because of the new prosumers that have been activated and grouped the VPP, whose total active power contribution is shown with the red line. 

**Figure 13:**
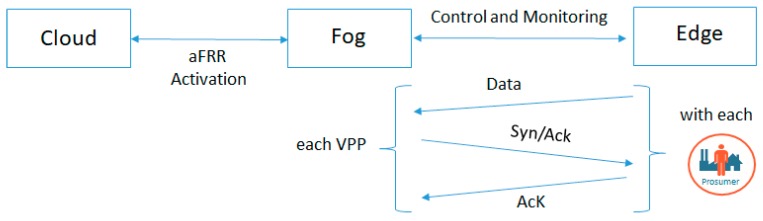
Messages exchange in our fog based approach.

Finally, we evaluate the benefits of using a fog-edge based architecture for data collection and virtual power plant dynamic coalition creation from the perspective of optimization problem complexity and the number of messages sent to the prosumers for services activation. We suppose that we have a monitoring system based on smart meters deployed at the prosumer side that have a sampling rate of 5 seconds. In the classical architecture, all the data is collected to a central point (i.e., in the cloud), while in our fog-edge architecture, data is stored at the microgrid level. At the same time, the aFRR activation message is now sent only once from the cloud level to the fog level where the VPPs are being created and only then distributed to all newly identified prosumers for their activation. Figure 13 presents the way in which the messages are being exchanged in our case, also following considerations provided in reference [44] for TCP/IP communication with energy resources. 

**Figure 14:**
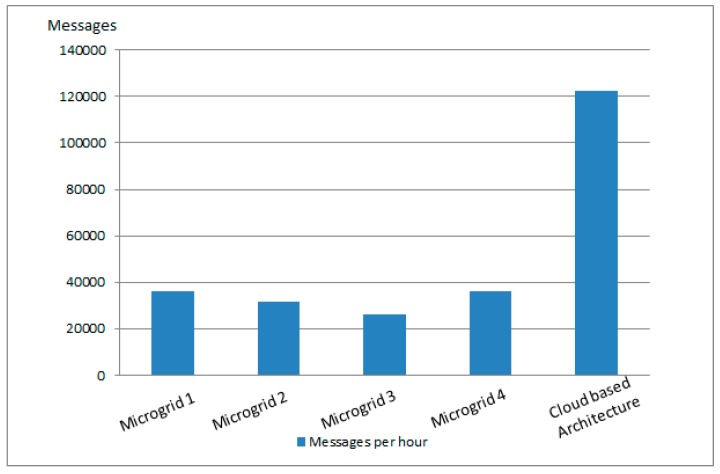
Number of messages sent during one hour by smart meters associated with prosumers.

As shown in Figure 14, the number of messages in the case of a decentralized control at fog and edge level decreases the number of data messages sent over the communication network, thereby decongesting the networks. 

Furthermore, the decentralized control also has an effect on the optimization problem, solving time and complexity. We analyze the impact of decentralization on the optimization problem solved using a genetic algorithm to determine an approximate solution. The solver was run on a platform using Intel I3, 16GB of RAM, and Ubuntu 18, and was configured with 100×N individuals (100 of times the number of prosumers from the portfolio) and a maximum number of iterations less than 100. 

**Figure 15:**
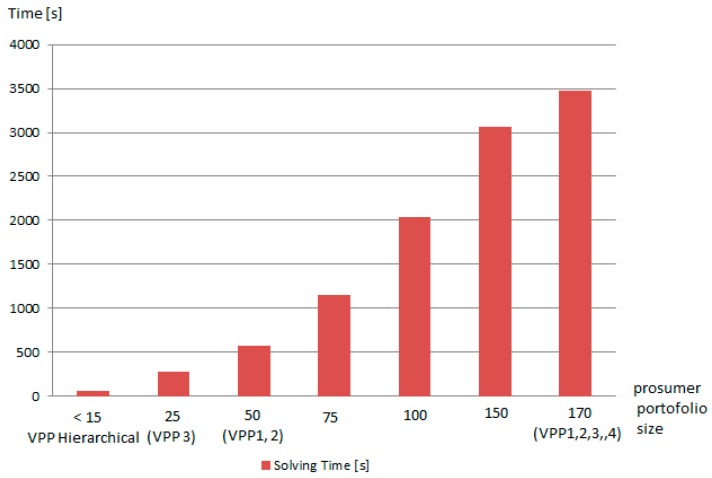
Time needed to solve the optimization problem for the VPP model in our scenario.

As shown in Figure 15, to determine the optimal coalition for a small number of prosumers such as when creating a hierarchical VPP in our scenario, the solving time is much smaller compared with the nonhierarchical approach. Moreover, the time needed to compute the solution is less than 10 minutes, making the model viable for addressing the aFRR service constraints (the time for solution generation in the nonhierarchical case is unfeasible for aFRR implementation).

## 6. Conclusions

This paper presents a fog computing-based infrastructure for smart energy grid management decentralization and a VPP model allowing the dynamic creation of coalitions of prosumers at the fog layer to deliver ancillary services to the main grid. The VPP model has been extended and specialized for the frequency restoration reserve ancillary service, which has strong response time constraints. The results carried out in a simulated environment show our approach’s feasibility to construct and manage virtual coalitions of prosumers on demand, by selecting and activating additional small scale prosumers to deliver active power at the microgrid level, allowing us to locally address potential unbalances and avoid having to escalate them to the main grid level. Moreover, after considering the experiments estimating the number of messages and the solving time of the optimization problem, we can conclude that a hierarchical VPP coalition formation is feasible even for frequency restoration reserve constraints because in this case any optimization problems with a smaller size can be solved more quickly. For future work, we aim to formalize the optimal coalition formation to other types of ancillary services and to investigate the potential of decentralization over blockchain-based infrastructure.

## Figures and Tables

**Table 1:** Experimental setup for prosumers generation.

Experimental Setup	Values
Prosumer Minimum Generation	0
Prosumer Maximum Generation	3.34 kWh
$U_{L}$	90%
$U_{H}$	110%

**Table 2:** Load-frequency control technical requirements [41].

Load-Frequency Control Action	Activation Time	Cycle Time
aFRR	5 min–15 min	1–5 s
